# Supplementary material for: Deuterium metabolic imaging phenotypes mouse glioblastoma heterogeneity through glucose turnover kinetics
Source: eLife. 2025 Mar 4;13:RP100570. doi: 10.7554/eLife.100570 (PMC11879113; doi:10.7554/eLife.100570)
Supplement: Supplementary file 1. [file elife-100570-supp1.docx]

**Supplementary file 1a - Table 1. Tumor cohorts.** Animal information and metrics obtained from multi-modal *in vivo* MRI and *post-mortem* histopathology and immunostaining.

|  |  | **CT2A TUMORS** | | | | | **CT2A COHORT** | | | **GL261 TUMORS** | | | | | **GL261 COHORT** | | | **GL261 vs CT2A** | | **POOLED COHORTS** | | |
| --- | --- | --- | --- | --- | --- | --- | --- | --- | --- | --- | --- | --- | --- | --- | --- | --- | --- | --- | --- | --- | --- | --- |
|  | **Parameter** | **C1** | **C2** | **C3** | **C4** | **C5** | **(mean±SE)** | | | **G1** | **G2** | **G3** | **G4** | **G5** | **(mean±SE)** | | | **(%)** | **(p) ^c^** | **(mean±SE)** | | |
| **Animal** | Batch | 1 | 2 | 2 | 2 | 2 | N.A. | | | 1 | 1 | 2 | 2 | 2 | N.A. | | | N.A. | N.A. | N.A. | | |
|  | Gender | male | male | male | male | male | N.A. | | | male | male | male | male | male | N.A. | | | N.A. | N.A. | N.A. | | |
|  | **Days PI** | 18 | 45 | 22 | 28 | 37 | 30 | ± | 5 | 18 | 18 | 16 | 17 | 17 | 17 | ± | 0 | **42.7%** | **0.03215** | 24 | ± | 3 |
|  | **Weight (g)** | 24.3 | 23.0 | 26.6 | 26.4 | 28.3 | 25.7 | ± | 0.9 | 22.1 | 24.5 | 21.9 | 20.8 | 22.7 | 22.4 | ± | 0.6 | **-12.9%** | **0.01741** | 24.1 | ± | 0.8 |
| **T2w MRI** | Tumor Volume (mm^3^) | 113.9 | 41.2 | 52.3 | 34.7 | 59.8 | 60.4 | ± | 14.1 | 41.0 | 49.1 | 74.6 | 64.6 | 54.1 | 56.7 | ± | 5.9 | -6.2% | 0.81283 | 58.5 | ± | 7.2 |
| **DGE-DMI** | WL (Hz) | 31.2 | 33.3 | 24.7 | 20.4 | 36.5 | 29.2 | ± | 2.9 | 20.4 | 22.6 | 27.9 | 29 | 30.1 | 26.0 | ± | 1.9 | -11.0% | 0.38367 | 27.6 | ± | 1.7 |
|  | SNRi orig (average) | 6.5 | 5.7 | 6.1 | 6.0 | 6.6 | 6.2 | ± | 0.2 | 6.2 | 6.9 | 6.7 | 6.9 | 6.1 | 6.6 | ± | 0.2 | 6.1% | 0.15013 | 6.4 | ± | 0.1 |
|  | SNRi den (average) | 20.4 | 18.3 | 19.5 | 18.7 | 20.4 | 19.5 | ± | 0.4 | 20.0 | 22.1 | 20.0 | 22.5 | 19.5 | 20.8 | ± | 0.6 | 7.0% | 0.10721 | 20.1 | ± | 0.4 |
|  | ***Tumor (ROI):*** |  |  |  |  |  |  |  |  |  |  |  |  |  |  |  |  |  |  |  |  |  |
|  | Glc (mM) ^a^ | 1.73 | 0.77 | 1.33 | 1.25 | 1.35 | 1.29 | ± | 0.15 | 1.69 | 1.64 | 1.71 | 2.13 | 1.28 | 1.69 | ± | 0.13 | 31.4% | 0.08249 | 1.49 | ± | 0.12 |
|  | Glx (mM) ^a^ | 0.71 | 0.04 | 0.82 | 0.79 | 0.72 | 0.62 | ± | 0.15 | 1.00 | 0.15 | 0.64 | 0.05 | 0.13 | 0.40 | ± | 0.18 | -35.5% | 0.37892 | 0.51 | ± | 0.12 |
|  | **Lac (mM) ^a^** | 4.06 | 2.22 | 2.46 | 2.61 | 3.22 | 2.91 | ± | 0.33 | 2.50 | 1.76 | 1.22 | 1.30 | 1.21 | 1.60 | ± | 0.25 | **-45.2%** | **0.01283** | 2.26 | ± | 0.29 |
|  | V_max_(mM.min^-1^) ^b^ | 0.951 | 0.512 | 0.645 | 0.590 | 0.828 | 0.705 | ± | 0.080 | 0.784 | 0.463 | 0.733 | 0.616 | 0.750 | 0.669 | ± | 0.059 | -5.1% | 0.72736 | 0.687 | ± | 0.047 |
|  | V_glx_ (mM.min^-1^) ^b^ | 0.273 | 0.159 | 0.195 | 0.142 | 0.262 | 0.207 | ± | 0.027 | 0.214 | 0.075 | 0.318 | 0.726 | 0.403 | 0.347 | ± | 0.109 | 68.1% | 0.24636 | 0.277 | ± | 0.058 |
|  | V_lac_ (mM.min^-1^) ^b^ | 0.818 | 0.484 | 0.488 | 0.495 | 0.685 | 0.594 | ± | 0.068 | 0.764 | 0.429 | 0.604 | 0.565 | 0.530 | 0.578 | ± | 0.055 | -2.6% | 0.86151 | 0.586 | ± | 0.041 |
|  | K_glx_ (mM.min^-1^) ^b^ | 0.114 | 0.051 | 0.073 | 0.035 | 0.093 | 0.073 | ± | 0.014 | 0.045 | 0.144 | 0.093 | 0.254 | 0.174 | 0.142 | ± | 0.036 | 94.4% | 0.11066 | 0.107 | ± | 0.021 |
|  | **K_lac_ (mM.min^-1^) ^b^** | 0.067 | 0.042 | 0.056 | 0.049 | 0.063 | 0.055 | ± | 0.005 | 0.088 | 0.068 | 0.136 | 0.136 | 0.110 | 0.108 | ± | 0.013 | **94.3%** | **0.00619** | 0.082 | ± | 0.011 |
|  | ***Peritumoral Rim (ROI):*** |  |  |  |  |  |  |  |  |  |  |  |  |  |  |  |  |  |  |  |  |  |
|  | Glc (mM) ^a^ | 2.227 | 0.898 | 1.699 | 1.785 | 1.737 | 1.67 | ± | 0.22 | 1.633 | 1.325 | 1.503 | 1.958 | 1.461 | 1.58 | ± | 0.11 | -5.6% | 0.70857 | 1.62 | ± | 0.11 |
|  | Glx (mM) ^a^ | 0.551 | 0.382 | 0.846 | 1.026 | 1.023 | 0.77 | ± | 0.13 | 1.477 | 0.472 | 0.812 | 0.561 | 0.698 | 0.80 | ± | 0.18 | 5.0% | 0.86554 | 0.78 | ± | 0.10 |
|  | **Lac (mM) ^a^** | 2.005 | 1.096 | 1.476 | 1.144 | 1.586 | 1.46 | ± | 0.17 | 1.272 | 0.976 | 0.755 | 0.871 | 0.840 | 0.94 | ± | 0.09 | **-35.5%** | **0.02466** | 1.20 | ± | 0.12 |
|  | V_max_(mM.min^-1^) ^b^ | 0.597 | 0.427 | 0.440 | 0.528 | 0.536 | 0.505 | ± | 0.032 | 0.621 | 0.286 | 0.426 | 0.717 | 0.746 | 0.559 | ± | 0.088 | 10.6% | 0.58343 | 0.532 | ± | 0.045 |
|  | V_glx_ (mM.min^-1^) ^b^ | 0.125 | 0.161 | 0.207 | 0.219 | 0.216 | 0.186 | ± | 0.018 | 0.306 | 0.060 | 0.171 | 0.326 | 0.611 | 0.295 | ± | 0.093 | 58.8% | 0.28113 | 0.240 | ± | 0.048 |
|  | V_lac_ (mM.min^-1^) ^b^ | 0.587 | 0.398 | 0.355 | 0.577 | 0.475 | 0.479 | ± | 0.047 | 0.557 | 0.240 | 0.391 | 0.548 | 0.313 | 0.410 | ± | 0.063 | -14.4% | 0.40435 | 0.444 | ± | 0.039 |
|  | K_glx_ (mM.min^-1^) ^b^ | 0.056 | 0.062 | 0.046 | 0.049 | 0.060 | 0.055 | ± | 0.003 | 0.038 | 0.027 | 0.051 | 0.114 | 0.187 | 0.083 | ± | 0.030 | 52.4% | 0.36967 | 0.069 | ± | 0.015 |
|  | K_lac_ (mM.min^-1^) ^b^ | 0.091 | 0.071 | 0.077 | 0.141 | 0.089 | 0.094 | ± | 0.012 | 0.109 | 0.060 | 0.120 | 0.240 | 0.094 | 0.124 | ± | 0.030 | 32.7% | 0.37862 | 0.109 | ± | 0.016 |
| **DCE-T1w MRI** | ***Tumor (ROI):*** |  |  |  |  |  |  |  |  |  |  |  |  |  |  |  |  |  |  |  |  |  |
|  | **K^trans^ (min^-1^)** | 0.00527 | 0.00288 | N.A. | 0.00465 | 0.00445 | 0.00431 | ± | 0.00051 | 0.00532 | 0.00947 | 0.00976 | N.A. | 0.00959 | 0.00854 | ± | 0.00107 | **97.9%** | **0.01199** | 0.00642 | ± | 0.00097 |
|  | K^ep^ (min^-1^) | 0.03455 | 0.03037 | N.A. | 0.03920 | 0.03059 | 0.03368 | ± | 0.00208 | 0.02128 | 0.03598 | 0.05167 | N.A. | 0.04561 | 0.03864 | ± | 0.00663 | 14.7% | 0.50174 | 0.03616 | ± | 0.00335 |
|  | v (%) | 0.192 | 0.140 | N.A. | 0.148 | 0.230 | 0.178 | ± | 0.021 | 0.346 | 0.272 | 0.211 | N.A. | 0.227 | 0.264 | ± | 0.030 | 48.7% | 0.05658 | 0.221 | ± | 0.024 |
|  | ***Peritumoral Rim (ROI):*** |  |  |  |  |  |  |  |  |  |  |  |  |  |  |  |  |  |  |  |  |  |
|  | K^trans^ (min^-1^) | 0.00294 | 0.00109 | N.A. | 0.00190 | 0.00084 | 0.00169 | ± | 0.00047 | 0.00116 | 0.00257 | 0.00240 | N.A. | 0.00230 | 0.00211 | ± | 0.00032 | 24.5% | 0.49496 | 0.00190 | ± | 0.00028 |
|  | K^ep^ (min^-1^) | 0.04049 | 0.04568 | N.A. | 0.01839 | 0.03472 | 0.03482 | ± | 0.00592 | 0.02515 | 0.05097 | 0.08493 | N.A. | 0.04581 | 0.05171 | ± | 0.01240 | 48.5% | 0.26486 | 0.04327 | ± | 0.00712 |
|  | v (%) | 0.085 | 0.026 | N.A. | 0.150 | 0.067 | 0.082 | ± | 0.026 | 0.105 | 0.106 | 0.026 | N.A. | 0.103 | 0.085 | ± | 0.020 | 3.3% | 0.93572 | 0.083 | ± | 0.015 |
| **Histology** | Phenotype (H&E score) | I | III | III | I | III | 0/5 ^d^ | | | IV | IV | IV | IV | IV | 5/5 ^d^ | | | N.A. | N.A. | N.A. | | |
|  | Infiltration (+ yes; ̶ no) | ‒ | ‒ | ‒ | ‒ | ‒ | 0/5 ^e^ | | | + | + | + | + | + | 5/5 ^e^ | | | N.A. | N.A. | N.A. | | |
|  | Distant Migration (+ yes; ̶ no) | + | ‒ | ‒ | ‒ | ‒ | 1/5 ^e^ | | | ‒ | ‒ | + | + | + | 3/5 ^e^ | | | N.A. | N.A. | N.A. | | |
|  | ***Tumor (ROI):*** |  |  |  |  |  |  |  |  |  |  |  |  |  |  |  |  |  |  |  |  |  |
|  | Area (10^7^ µm^2^) | 1.70 | 0.84 | 0.80 | 1.02 | 1.15 | 1.10 | ± | 0.16 | 1.11 | 1.24 | 1.59 | 4.01 | 1.15 | 1.82 | ± | 0.56 | 65.1% | 0.24978 | 1.46 | ± | 0.30 |
|  | Cellularity (10^5^ cells) | 1.20 | 0.67 | 0.69 | 0.94 | 0.96 | 0.89 | ± | 0.10 | 0.56 | 0.60 | 0.85 | 0.66 | 0.47 | 0.63 | ± | 0.06 | -29.8% | 0.05224 | 0.76 | ± | 0.07 |
|  | **Cell Density (10^-3^ cells/um^2^)** | 7.07 | 8.10 | 8.24 | 9.21 | 8.45 | 8.22 | ± | 0.34 | 5.06 | 4.81 | 5.35 | 5.17 | 4.07 | 4.89 | ± | 0.22 | **-40.4%** | **0.00004** | 6.56 | ± | 0.59 |
|  | Cell Proliferation (Ki67^+^ %) | 52.2 | 65.0 | 61.7 | 72.8 | 68.7 | 64.1 | ± | 3.5 | 76.8 | 77.8 | 76.1 | 70.0 | 62.1 | 72.5 | ± | 2.9 | 13.2% | 0.10187 | 68.3 | ± | 2.6 |
|  | Mgl/Mp infiltration (% area) | 2.8 |  | 4.7 | 7.9 | 1.4 | 4.2 | ± | 1.4 | 3.0 | 0.6 | 0.1 | 2.6 | 2.2 | 1.7 | ± | 0.6 | -58.9% | 0.12320 | 2.8 | ± | 0.8 |
|  | ***Peritumoral Rim (ROI):*** |  |  |  |  |  |  |  |  |  |  |  |  |  |  |  |  |  |  |  |  |  |
|  | Area (10^7^ µm^2^) | 0.45 | 0.33 | 0.25 | 0.36 | 0.34 | 0.35 | ± | 0.03 | 0.33 | 0.32 | 0.30 | 0.31 | 0.31 | 0.31 | ± | 0.01 | -9.3% | 0.35993 | 0.33 | ± | 0.02 |
|  | Cellularity (10^5^ cells) | 0.08 | 0.07 | 0.04 | 0.06 | 0.06 | 0.06 | ± | 0.01 | 0.05 | 0.05 | 0.06 | 0.17 | 0.06 | 0.08 | ± | 0.02 | 20.9% | 0.59409 | 0.07 | ± | 0.01 |
|  | Cell Density (10^-3^ cells/um^2^) | 1.87 | 2.04 | 1.77 | 1.84 | 1.64 | 1.83 | ± | 0.07 | 1.55 | 1.48 | 2.03 | 6.91 | 1.89 | 2.77 | ± | 1.04 | 51.4% | 0.39236 | 2.30 | ± | 0.52 |
|  | Cell Proliferation (Ki67^+^ %) | 16.6 | 27.8 | 25.5 | 24.3 | 17.4 | 22.3 | ± | 2.3 | 18.4 | 20.5 | 16.7 | 18.8 | 18.2 | 18.5 | ± | 0.6 | -17.0% | 0.14291 | 20.4 | ± | 1.3 |
|  | **Mgl/Mp infiltration (% area)** | 8.7 |  | 15.7 | 10.9 | 4.8 | 10.0 | ± | 2.3 | 4.5 | 1.5 | 0.2 | 1.6 | 2.9 | 2.1 | ± | 0.7 | **-78.6%** | **0.00839** | 5.6 | ± | 1.7 |

*K^trans^*, volume transfer constant between plasma and tumor extravascular-extracellular space; *k_ep_*, washout rate between extravascular-extracellular space and plasma; *v_e_*, extravascular-extracellular volume fraction; WL, water linewidth at half-maximum of the water peak; *Vglx*, maximum rate of Glc consumption for Glx synthesis (mM. min−1); *Vlac*, maximum rate of Glc consumption for Lac synthesis (mM. min−1); *Vmax*, maximum rate of total Glc consumption (mM. min−1); ^a^ Temporal average (post-injection); ^b^ Kinetic flux rate; ^c^ 2-tailed, unpaired t-Test (highlighted p<0.05); ^d^ tumors with score IV (Supplementary Table 2); ^e^ tumors with infiltration, migration, or both.

**Supplementary file 1b - Table 2. Histopathologic evaluation.** Analysis of H&E histologic sections from each tumor and scoring according to phenotypic features of their stromal-vascular fraction.

| **SAMPLE** | **TUMOR** | | | | | | | | **VESSELS** | | | **BRAIN** | | | **RESULTS** |
| --- | --- | --- | --- | --- | --- | --- | --- | --- | --- | --- | --- | --- | --- | --- | --- |
| **ID** | **primary location** | **secondary location** | **growth pattern^a^** | **tumor cell morphology** | **cell-cell interaction** | **stroma** | **tumor border^a^** | **tumor necrosis** | **density** | **instability** | **mimicry** | **vascular invasion^b^** | **intercallation** | **brain damage** | **Phenotype score^c^** |
| **C1** | cerebral nuclei: caudoputamen | midbrain | expansile | large, poligonal to spindle | cohesive | non-cystic, non-oedematous, non-hemorrhagic | outward pushing | minimal | moderate | mild | not evident | not evident | 0 or minimal | minimal: compressive, ischemic | **I** |
| **C2** | cerebral nuclei: caudoputamen | none | expansile | large, poligonal to spindle | poorly cohesive | cystic, oedematous, hemorrhagic | outward pushing | moderate | moderate | marked | present | not evident | mild | mild: compressive, ischemic | **III** |
| **C3** | cerebral nuclei: caudoputamen | none | expansile | large, poligonal to spindle | cohesive | non-cystic, non-oedematous, poorly hemorrhagic | outward pushing | mild | moderate | moderate | present | not evident | 0 or minimal | minimal: compressive, ischemic | **III** |
| **C4** | cerebral nuclei: caudoputamen | none | expansile | large, poligonal to spindle | cohesive | non-cystic, non-oedematous, non-hemorrhagic | outward pushing | minimal | moderate | mild | not evident | not evident | 0 or minimal | minimal: compressive, ischemic | **I** |
| **C5** | cerebral nuclei: caudoputamen | none | expansile | large, spindle | poorly cohesive | cystic, oedematous, hemorrhagic | outward pushing | moderate | moderate | marked | present | not evident | mild | mild: compressive, hemorrhagic | **III** |
| **G1** | cerebral nuclei: caudoputamen | none | expansile, infiltrative | large, poligonal, multinucleated | poorly cohesive | cystic, oedematous, non-hemorrhagic | outward pushing and solid strand collective migration | minimal | low | mild | present | not evident | moderate | minimal: compressive | **IV** |
| **G2** | cerebral nuclei: caudoputamen | none | expansile, infiltrative | large, poligonal, multinucleated | poorly cohesive | cystic, oedematous, non-hemorrhagic | outward pushing and solid strand collective migration | minimal | low | mild | present | not evident | moderate | minimal: compressive | **IV** |
| **G3** | cerebral nuclei: caudoputamen | infiltrating ipsiateral and contralateral lateral ventricle | expansile | large, poligonal, multinucleated | poorly cohesive at 1ary tumor | cystic, oedematous, non-hemorrhagic | outward pushing | minimal | moderate | moderate | present | not evident | 0 or minimal | minimal: compressive, ischemic | **IV** |
| **G4** | cerebral nuclei: caudoputamen | infiltrating ipsiateral and contralateral lateral ventricle | expansile | large, poligonal, multinucleated | non-cohesive | cystic, oedematous, hemorrhagic | outward pushing and solid strand collective migration | minimal | moderate | marked | present | not evident | 0 or minimal | minimal: compressive, ischemic | **IV** |
| **G5** | cerebral nuclei: caudoputamen | infiltrating ipsiateral lateral ventricle | expansile | large, poligonal, multinucleated | non-cohesive | cystic, oedematous, hemorrhagic | outward pushing and solid strand collective migration | minimal | moderate | marked | present | not evident | 0 or minimal | minimal: compressive, ischemic | **IV** |

a predominant; b 0 (absent) or 1 (present); c progression phase; d tumors studied *in vivo* under acute hypoxia.

**Supplementary file 1c - Table 3. Tumor-to-border ratios in CT2A and GL261 cohorts.** Animal information and metrics obtained from multi-modal *in vivo* MRI and *post-mortem* histopathology and immunostaining. Initial values taken from Supplementary table 1.

|  |  | **CT2A TUMORS** | | | | | **CT2A COHORT** | | | **GL261 TUMORS** | | | | | **GL261 COHORT** | | | **CT2A vs GL261** | | **POOLED COHORTS** | | |
| --- | --- | --- | --- | --- | --- | --- | --- | --- | --- | --- | --- | --- | --- | --- | --- | --- | --- | --- | --- | --- | --- | --- |
|  | **Parameter** | **C1** | **C2** | **C3** | **C4** | **C5** | **(mean±SE)** | | | **G1** | **G2** | **G3** | **G4** | **G5** | **(mean±SE)** | | | **(%)** | **(p) ^c^** | **(mean±SE)** | | |
| **DGE-DMI** | ***Tumor/PT-Rim (ROI):*** |  |  |  |  |  |  |  |  |  |  |  |  |  |  |  |  |  |  |  |  |  |
|  | **Glc ^a^** | 0.78 | 0.86 | 0.78 | 0.70 | 0.78 | 0.78 | ± | 0.02 | 1.03 | 1.24 | 1.14 | 1.09 | 0.88 | 1.08 | ± | 0.06 | **37.9%** | **0.00185** | 0.93 | ± | 0.06 |
|  | Glx ^a^ | 1.29 | 0.10 | 0.98 | 0.77 | 0.70 | 0.77 | ± | 0.20 | 0.68 | 0.32 | 0.79 | 0.10 | 0.19 | 0.42 | ± | 0.14 | -45.8% | 0.17812 | 0.59 | ± | 0.13 |
|  | **Lac ^a^** | 2.03 | 2.03 | 1.67 | 2.28 | 2.03 | 2.01 | ± | 0.10 | 1.97 | 1.80 | 1.61 | 1.49 | 1.44 | 1.66 | ± | 0.10 | **-17.1%** | **0.03814** | 1.83 | ± | 0.09 |
|  | V_max_ ^b^ | 1.59 | 1.20 | 1.47 | 1.12 | 1.54 | 1.38 | ± | 0.10 | 1.26 | 1.62 | 1.72 | 0.86 | 1.01 | 1.29 | ± | 0.17 | -6.5% | 0.65134 | 1.340 | ± | 0.092 |
|  | V_glx_ ^b^ | 2.18 | 0.99 | 0.94 | 0.65 | 1.21 | 1.20 | ± | 0.26 | 0.70 | 1.24 | 1.86 | 2.22 | 0.66 | 1.34 | ± | 0.31 | 11.9% | 0.73566 | 1.266 | ± | 0.193 |
|  | V_lac_ ^b^ | 1.39 | 1.22 | 1.38 | 0.86 | 1.44 | 1.26 | ± | 0.11 | 1.37 | 1.79 | 1.54 | 1.03 | 1.70 | 1.49 | ± | 0.13 | 18.3% | 0.21603 | 1.371 | ± | 0.089 |
|  | K_glx_ ^b^ | 2.03 | 0.81 | 1.60 | 0.70 | 1.54 | 1.34 | ± | 0.25 | 1.18 | 5.38 | 1.81 | 2.22 | 0.93 | 2.31 | ± | 0.80 | 72.4% | 0.28244 | 1.821 | ± | 0.428 |
|  | **K_lac_ ^b^** | 0.74 | 0.59 | 0.73 | 0.35 | 0.71 | 0.62 | ± | 0.07 | 0.80 | 1.13 | 1.14 | 0.57 | 1.17 | 0.96 | ± | 0.12 | **54.9%** | **0.04011** | 0.792 | ± | 0.087 |
| **DCE-T1w MRI** | ***Tumor/PT-Rim (ROI):*** |  |  |  |  |  |  |  |  |  |  |  |  |  |  |  |  |  |  |  |  |  |
|  | K^trans^ | 1.79254 | 2.64653 | N.A. | 2.45347 | 5.27242 | 3.04124 | ± | 0.76587 | 4.58606 | 3.67981 | 4.06757 | N.A. | 4.17236 | 4.12645 | ± | 0.18626 | 35.7% | 0.21773 | 3.58385 | ± | 0.41855 |
|  | K^ep^ | 0.85324 | 0.66484 | N.A. | 2.13101 | 0.88103 | 1.13253 | ± | 0.33627 | 0.84638 | 0.70592 | 0.60833 | N.A. | 0.99583 | 0.78911 | ± | 0.08447 | -30.3% | 0.36019 | 0.96082 | ± | 0.17312 |
|  | v | 2.25528 | 5.42510 | N.A. | 0.98829 | 3.41765 | 3.022 | ± | 0.942 | 3.29250 | 2.58069 | 8.17438 | N.A. | 2.20769 | 4.064 | ± | 1.389 | 34.5% | 0.55739 | 3.543 | ± | 0.801 |
| **Histology** | ***Tumor/PT-Rim (ROI):*** |  |  |  |  |  |  |  |  |  |  |  |  |  |  |  |  |  |  |  |  |  |
|  | Area | 3.76 | 2.56 | 3.23 | 2.81 | 3.37 | 3.15 | ± | 0.21 | 3.31 | 3.89 | 5.32 | 12.78 | 3.77 | 5.81 | ± | 1.77 | 84.8% | 0.17363 | 4.48 | ± | 0.95 |
|  | Cellularity | 14.17 | 9.84 | 15.42 | 14.41 | 17.28 | 14.22 | ± | 1.22 | 11.07 | 12.61 | 13.98 | 3.89 | 8.13 | 9.93 | ± | 1.80 | -30.2% | 0.08391 | 12.08 | ± | 1.25 |
|  | **Cell Density** | 3.78 | 3.97 | 4.66 | 5.00 | 5.16 | 4.51 | ± | 0.27 | 3.26 | 3.25 | 2.63 | 0.75 | 2.15 | 2.41 | ± | 0.46 | **-46.6%** | **0.00453** | 3.46 | ± | 0.43 |
|  | **Cell Proliferation** | 3.2 | 2.3 | 2.4 | 3.0 | 3.9 | 3.0 | ± | 0.3 | 4.2 | 3.8 | 4.6 | 3.7 | 3.4 | 3.9 | ± | 0.2 | **32.3%** | **0.02568** | 3.5 | ± | 0.2 |
|  | Mgl/Mp infiltration (% area) | 0.3 | N.A. | 0.3 | 0.7 | 0.3 | 0.4 | ± | 0.1 | 0.7 | 0.4 | 0.5 | 1.6 | 0.8 | 0.8 | ± | 0.2 | 96.6% | 0.18177 | 0.6 | ± | 0.1 |

*K^trans^*, volume transfer constant between plasma and tumor extravascular-extracellular space; *k_ep_*, washout rate between extravascular-extracellular space and plasma; *v_e_*, extravascular-extracellular volume fraction; WL, water linewidth at half-maximum of the water peak; *Vglx*, maximum rate of Glc consumption for Glx synthesis (mM. min−1); *Vlac*, maximum rate of Glc consumption for Lac synthesis (mM. min−1); *Vmax*, maximum rate of total Glc consumption (mM. min−1); ^a^ Temporal average (post-injection); ^b^ Kinetic flux rate; ^c^ 2-tailed, unpaired t-Test (highlighted p<0.05).
